# Annotations

**Published:** 1897-03-27

**Authors:** 


### Annotations.

### What is a "Medical Charity"?

Most of us entertain an opinion in a general way that we know quite well what a "medical charity" is. But Dr. Campbell Black has published a definition in a Scotch newspaper; and if he is right we are all very far wrong indeed. The "end and aim" of a medical charity, Dr. Black tells us, is to make "consulting physicians and surgeons," and "it is for this purpose nowadays that all hospitals, great and small, special and general, exist." According to this definition there is no "charity" at all connected with hospitals. But again Dr. Black comes to our aid. There is a form of charity even at hospitals, he assures us; but the "charity is towards their founders and officials." Well now, if we must be quite plain—and in practical affairs it is "bad business" to be anything but plain—this is all arrant nonsense. Everybody, nowadays at any rate is satisfied that the out-patient departments of hospitals are abused; and everybody is anxious for a remedy. But is "scolding" any remedy? What remedy has Dr. Black for out-patient abuse? His only resource seems to be to proclaim a pessimistic unbelief in the capacity of the Hospital Reform Association. Now at the present moment there is a really great opportunity opening before hospital reformers of every class. The Prince of Wales's Jubilee Fund promises to be a magnificent success. As the result of this success, not only will there be a new administrative body created, but an amount of general and statesmanlike interest will be awakened in the subject, which, if it can but be rightly directed, may put an end not only to out-patient abuse, but to other abuses also. We have already urged the importance and the possibilities which attach to the Prince's Fund; and we look forward with much anxiety but also with much hope, to the use which medical men and hospital managers will put their coming opportunities.

### Food and Evolution.

The question has often been raised, What is the natural diet of man? and almost as often has it been answered that, by whatever long process of evolution the result has been arrived at, man is now constructed to be a mixed but largely a vegetable feeder, as shown both by his anatomy and physiology. By some it is considered that with this verdict the last word has been said; and we are not entirely unprepared to admit that if man has arrived at the acme of his possible perfection (a very gloomy and pessimistic suggestion), it might be best that he should keep to the hardy fare on which he has worked up to his present status. We are not, however, quite ready to allow that man has no future higher than the present, and we cannot but think that the alteration in man's food which the resources of civilisation are bringing about may, in another thousand years or so, make him a very different creature from what he hitherto has been. It becomes then a matter of considerable interest to inquire how far his anatomical and physiological attributes will allow of his assimilating the good things poured at his feet in such variety by the complex organisations of modern commerce. In regard to this it is interesting to observe how, under fresh circumstances, other animals will accept and even seek for diets which would appear quite unfitted for creatures of their anatomical construction. We have lately been told in the *Spectator* that in certain districts in Australia sheep have become not only carnivorous but cannibal, attacking the new-born lambs; that the English tame pigeon will now eat earth-worms on lawns as eagerly as a thrush; that with the decreasing amount of arable land, rooks have taken to eating young rabbits, pheasants, and chickens; and that on the other hand, in the presence of plenty of corn in the United States, the sparrow is giving up its insectivorous habits, and is becoming more and more dependent upon grain. Clearly there is a great range in the food that each animal can take. The question s, does the animal under the new food remain the same, and, if not, does it advance or degenerate? And, as to man, will he when brought up on the extraordinary variety of tasty morsels now brought for him from every quarter of the globe be so self-reliant, so hardy, and so well-fitted to conquer the world as he has been when reared on the simple food affected by Englishmen during past centuries?

### Schools and Diphtheria.

The relation between schools and diphtheria seems to be assuming a definiteness which it is difficult to gainsay. At the meeting of the London County Council last Tuesday, the reports of the Council's officials and that of the medical officer to the School Board, both of which we have already commented upon, came up for discussion, and along with them a further memorandum on a local prevalence of diphtheria in Camberwell was presented by Mr. Shirley Murphy, the facts detailed in which all went to confirm the view that schools exercise a not inconsiderable influence in the dissemination of the disease. Not only has Camberwell had a higher diphtheria rate for the past three years than has obtained throughout the rest of London; but a certain limited area in Camberwell, inhabited by about 20,000 people, has had a higher rate still, so that in 1896, while the London diphtheria case rate was 3·1, that of Camberwell (less the affected area) was 4·8, and that of the affected area was 14·6 per 1,000. Now, if the incidence of the disease on people of different ages is considered it is seen that, although all over London diphtheria specially affects children of school age, this epidemic in Camberwell attacked children of this age in higher proportion still. While children between 0 and 3 years of age in the affected area suffered 306 per cent. more than children of the same age in other parts of London, children between 3 and 5 suffered 359, those between 5 and 10 years of age 606, and those between 10 and 15 years of age 589 per cent. more than the children of the same ages in other parts of London, while in persons above 15 years of age the increase in the affected area fell again to only 282 above those of that age in the rest of London; so that it is clear that in this outbreak, as in so many others, children of school age were affected out of all proportion to those either younger or older than themselves. How far the schools are at fault is another matter. What seems clear is that, as at present managed, one of the penalties of the modern school system is that it conduces in no small degree to the dissemination of infectious diseases.

